# Incidentally Found Cholecystoduodenal Fistula and an Unusual Case of Gallstone Ileus After Laparoscopic Cholecystectomy

**DOI:** 10.7759/cureus.49651

**Published:** 2023-11-29

**Authors:** Saud Alsairy, Abdullah M Alessa, Bader Nasser Alaiyar, Osama Alharbi, Abdulaziz Alomar, Sakhar Albalawi, Bader Almalki, Ahmed AlRikhaimi

**Affiliations:** 1 Surgery, Security Forces Hospital, Riyadh, SAU; 2 Surgery, King Saud University, Riyadh, SAU; 3 Surgery, Imam Mohammad Ibn Saud Islamic University, Riyadh, SAU

**Keywords:** surgical case reports, acute care surgery, laparoscopic cholecystectomy, cholecystoduodenal fistula, gallstone ileus

## Abstract

Gallstone ileus, a rare and potentially fatal complication of cholelithiasis, occurs when gallstones breach the gastrointestinal tract through a fistula, causing an obstruction and potentially leading to severe complications. This case report details the experience of a 44-year-old woman with gallstone ileus stemming from an unnoticed cholecystoduodenal fistula following a routine cholecystectomy. The fistula was only discovered during surgery despite advanced imaging, revealing extensive adhesions. The discovery led to a subtotal cholecystectomy and fistula repair. Postoperatively, complications arose, prompting a computed tomography scan to rule out further issues. However, she later returned with gallstone ileus, necessitating a second operation. This case underscores the importance of thorough intraoperative exploration for biliary enteric fistulas during cholecystectomy, potentially averting the need for subsequent interventions. The case also highlights the diagnostic challenges of gallstone ileus and the significance of clinical suspicion.

## Introduction

Gallstone ileus is a rare and often late complication of cholelithiasis resulting from gallstones breaching the gastrointestinal (GI) tract through a fistula. The complication results in a small bowel obstruction. Courvoisier first described gallstone ileus as a late complication of cholecystitis in 1890 [1].

Although infrequent, gallstone ileus presents as a severe mechanical bowel obstruction in patients under 65. The sequelae of complications represent mechanical intestinal blockage or Bouveret’s syndrome. This syndrome affects its postoperative and intraoperative course, contributing to a reported 12% to 30% mortality rate [1-3]. For such a surgical disease, clinical symptoms are not specific. High clinical suspicion is warranted, followed by appropriate imaging. A computed tomography (CT) scan is the gold standard method for diagnosis, and surgical intervention includes dissection of the inflammatory adhesions, cholecystectomy, and en bloc resection of the fistula [1,2]. We report a case of a 44-year-old woman with a presentation of gallstone ileus with a missed cholecystoduodenal fistula after elective cholecystectomy. We describe the clinical presentation, diagnosis, surgical management, and postoperative course.

## Case presentation

A 44-year-old woman with an unremarkable medical and surgical background presented to the emergency department (ED) with a three-day history of right upper quadrant abdominal pain associated with constipation, persistent biliary vomiting aggravated by feeding, and nausea without fever. Ultrasonography revealed a 0.7-cm-dilated common bile duct, multiple stones within the gallbladder with a thick wall, and a rim of pericholecystic fluid. The patient was admitted for observation and conservative management and kept on nothing by mouth (NPO). On day three, an MRCP was conducted to rule out CBD stones, which were unremarkable. This patient did not tolerate the diet’s progression and persisted with symptoms of constipation. We took this patient for laparoscopic cholecystectomy and discovered many adhesions surrounding the inflamed gallbladder. Pus gushed out after we punctuated the fundus during adhesiolysis. Upon suctioning, we noticed a fistula between the gallbladder and the first part of the duodenum. We inserted an additional 15-mm port and cannulated the duct. An intraoperative cholangiogram revealed a long cystic duct with multiple filling defects that could likely be gas lobules rather than stones. Subtotal cholecystectomy was performed using Thick Tri-Staple endoGIA 45 mm.

Additionally, the fistula was dissected, and primary repair of the duodenum was achieved using a 2-0 V-lock suture. Over the repair, an omental patch was applied using interrupted size 3-0 vicryl stitches. Methylene blue dye was injected into the duodenum through a calibration tube and showed no intraoperative leak. A drain with a diameter of 19 Fr was inserted into the abdominal cavity, hemostasis was secured, ports were closed with the Endo Close device, and the skin was clipped shut. The patient was then transferred to recovery and later shifted to the ward.

**Figure 1:**
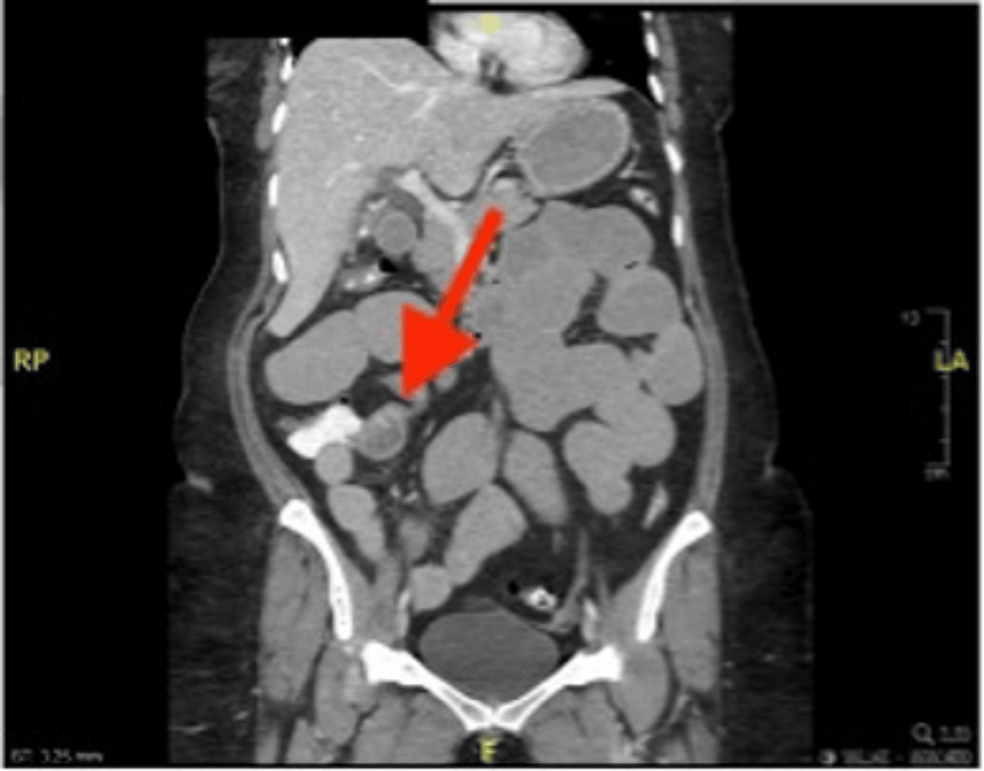
Coronal CT scan with contrast demonstrates the gallstone causing the ileus

**Figure 2:**
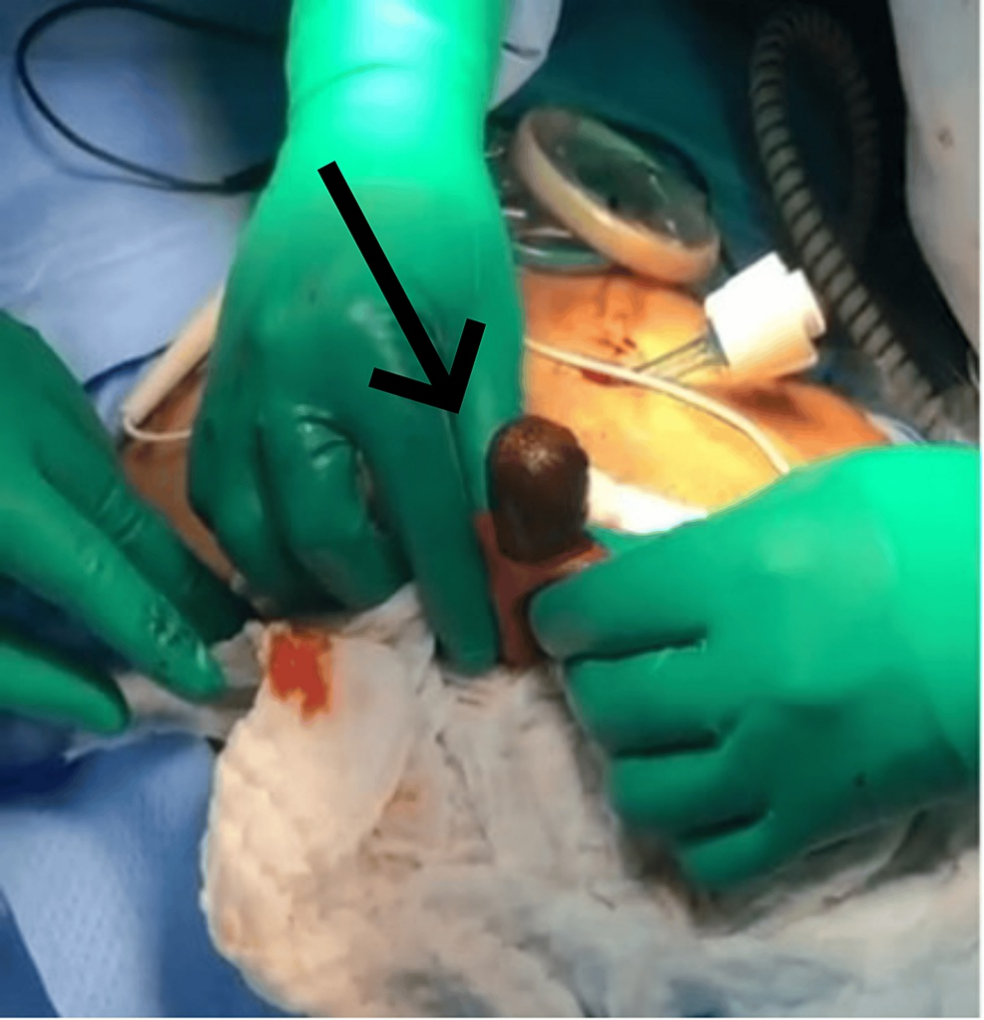
Intraoperative view of gallstone causing the ileus at the enterotomy site

**Figure 3:**
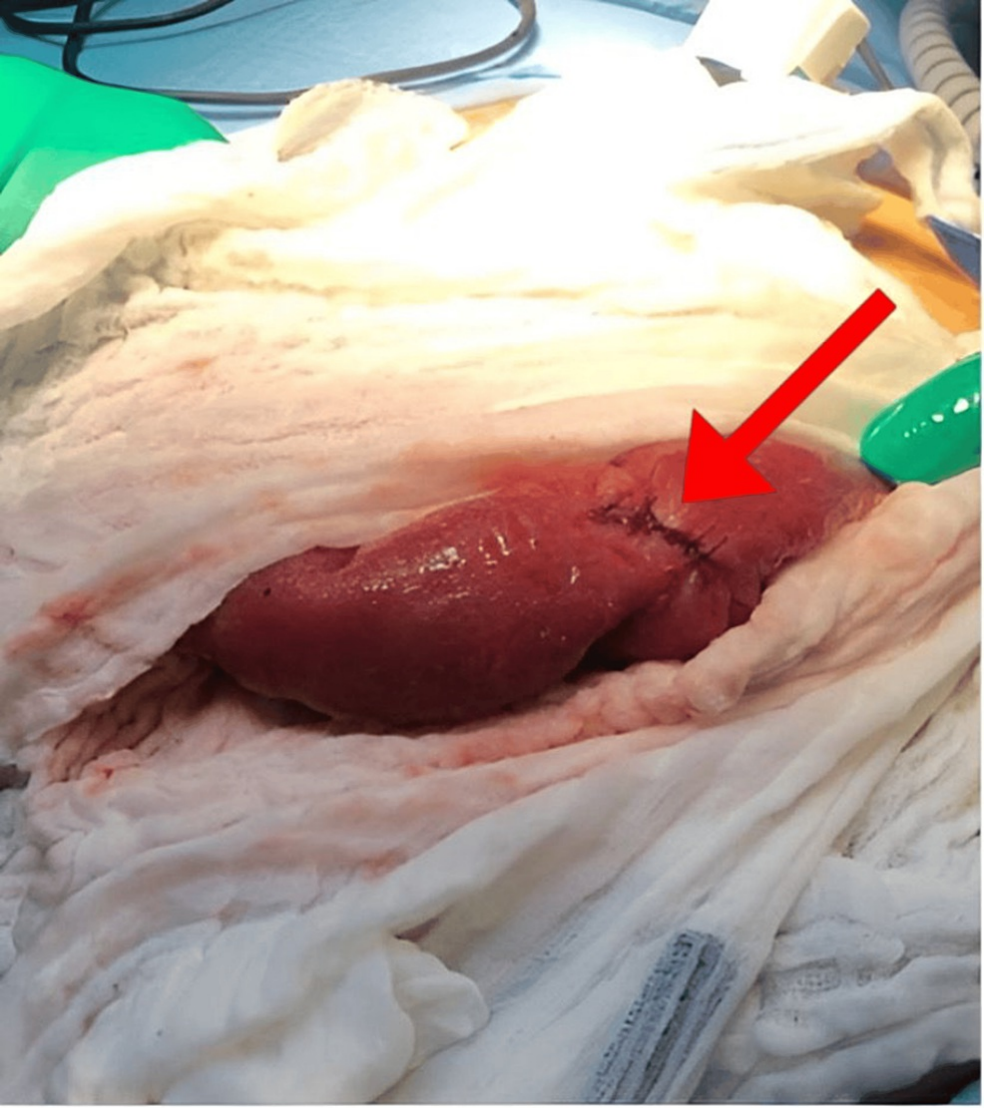
Intraoperative view showing enterotomy repair and the suture line

After surgery, on day two, the patient underwent a gastrografin study, which revealed the expected postsurgical narrowing of the first part of the duodenum without any evidence of contrast leakage into the proximal portion of the bowel. During the first three days postoperatively, her condition did not improve. She had an increasing leukocyte count, persistent nausea and vomiting, and intolerance of an oral diet. On postoperative day four, a CT scan of the abdomen and pelvis with IV contrast was performed to rule out any collections or bowel perforation, confirming the findings from the gastrografin study. The patient was discharged home after tolerating the oral diet, and her nausea and vomiting had subsided. Three days after discharge, she presented to the ED complaining of abdominal pain and persistent vomiting. The CT scan was reviewed again and showed a gallstone at the ileocecal valve (Figure 1). The patient was taken to the operating room, where an enterotomy (Figure 2-3) was performed, and the gallstone was extracted. She improved clinically and was discharged home a few days later.

## Discussion

Gallstone ileus, a rare complication of cholelithiasis, is an uncommon cause of mechanical bowel obstruction in 1-4% of patients younger than 65 [1]. Typically, the complication affects women and elderly patients [4-6], and the mortality rate ranges from 6.7% to 22.7% [7]. Recurrent inflammatory episodes in a gallbladder with stones are thought to be the pathophysiological source of adhesions to the surrounding tissues, particularly the GI tract. Gallstones can cause an increase in gallbladder pressure, resulting in decreased arterial and venous blood flow. Additionally, gallstones result in mucosal inflammation, ischemia, and gallbladder wall erosion. A fistula develops as the gallstone progresses toward the adherent intestinal segment [1,3,8].

Gallstones enter the digestive tract by cholecystoenteric fistulas in more than 90% of cases, with the cholecystoduodenal fistula being the most common [8]. It is also important to remember that not all people with cholecystoenteric fistulas experience GI symptoms. González Urquija et al. [2] reported 33.3% of the incidence in their series (n = 15), while Tandon et al. [9] reported 8.6% (n = 35). The stone’s size and the luminal diameter are the primary factors determining if an impact will occur [8]. For instance, unless the patient has a history of a condition resulting in intestinal lumen restriction, most stones less than 2 cm could move through a normal gastrointestinal system on their own. However, stones more than 2.5 cm are most susceptible to impact. Obstructing gallstones typically measure 4 cm in diameter [2,8], and 69% of cases are due to a single stone. The likelihood of recurrence increases when the obstruction is caused by cylindrical or faceted stones [8]. The most common gallstone impaction site is the ileum (50.0-60.5%), followed by the jejunum (16.1-26.9%), duodenum (3.5-14.6%), and the colon (3.0-4.1%) [8]. A consequence of gallstone impaction is Bouveret’s syndrome, a rare variant in the proximal duodenum or distal stomach, resulting in gastric outlet obstruction [2,8].

When performing a cholecystectomy, the presence of a cholecystoenteric fistula-documented in 1.4% of cases-should raise the possibility of GI complications, particularly when there is evidence of a stone, as there was in this case [9]. Unfortunately, the diagnosis of gallstone ileus is still overlooked or delayed, with only 77% of cases receiving an accurate preoperative diagnosis. The traditional radiological triad for this diagnosis includes pneumobilia, an ectopic gallstone, and mechanical intestinal blockage. A more precise diagnosis can be made because of the direct visualization of cholecystoenteric fistulae made possible by modern diagnostic imaging technologies, such as multidetector CT [8]. According to Gonzalez-Urquijo et al. [10], imaging modalities have recently been aiding the diagnosis, though only in 8-17% of cases. The MRCP showed the stone in this case, but the radiologist missed it. Our patient complained of right upper quadrant pain with nausea and vomiting but had no obvious signs of obstruction upon presentation. It was initially reported that when the internal bowel fistula was diagnosed during laparoscopic cholecystectomy, it carried a higher conversion rate to laparotomy [10,11]. We continued our operation laparoscopically and performed an intraoperative cholangiogram to confirm that the common bile duct was free of filling defects. A fistulectomy was done, and primary repair of the duodenum was achieved. However, postoperatively, the patient’s condition was not improving. Our suspicion at the time was a perforated viscus versus an intra-abdominal collection due to the severity of the inflammation found during the operation. However, the postoperative CT scan ruled out the presence of collection and perforation. The radiologists could not appreciate the presence of the gallstone. We attribute the worsening of symptoms postoperatively to the edema of the bowel loops resulting from bowel manipulation during the cholecystectomy. Three days after discharging the patient home, she returned to the ED with increased abdominal pain and vomiting frequency. The radiology department reassessed this patient’s CT images to confirm the diagnosis of gallstone ileus (Figure 2). We opted for a laparoscopic exploration and found a stone impacted at the terminal ileum, which was milked proximally, and a 10 cm midline incision was made to externalize the bowel. An enterotomy was done to extract the stone (Figure 3). Although a CT scan is the gold standard of diagnosis, subjective factors such as the radiologist’s level of experience could contribute to missed diagnosis. As surgeons, we encourage raising the suspicion and running the bowel to the level of the terminal ileum when a biliary enteric fistula is found incidentally during a cholecystectomy. Extracting the stone during the initial operation would have saved the patient from a second operation.

## Conclusions

In our clinical case report, we incidentally discovered a cholecystoduodenal fistula despite utilizing proper imaging modalities. Given the high likelihood of incidentally finding biliary enteric fistulas during laparoscopic cholecystectomy, we recommend intraoperatively examining the bowel to the level of the ileocecal valve. Performing such an examination could have saved the patient from requiring a second operation to extract the gallstone and alleviate the obstruction, thereby optimizing her treatment outcome.
